# Gaining information about home visits in primary care: methodological issues from a feasibility study

**DOI:** 10.1186/1471-2296-15-87

**Published:** 2014-05-06

**Authors:** Karen Voigt, Stephanie Taché, Andreas Klement, Thomas Fankhaenel, Stefan Bojanowski, Antje Bergmann

**Affiliations:** 1 Department of General Practice/Medical Clinic III, University Hospital Carl Gustav Carus of the Technische Universität Dresden, Fetscherstraße 74, 01307 Dresden, Germany; 2 Department of Family and Community Medicine, University California San Francisco, San Francisco, CA, USA; 3 Department of General Practice, Martin-Luther-University of Halle/Wittenberg, Halle, Germany

**Keywords:** Research design, Feasibility, General practice, Home visits

## Abstract

**Background:**

Home visits are part of general practice work in Germany. Within the context of an expanding elderly population and a decreasing number of general practitioner (GPs), open questions regarding the organisation and adequacy of GPs’ care in immobile patients remain. To answer these questions, we will conduct a representative primary data collection concerning contents and organisation of GPs’ home visits in 2014. Because this study will require considerable efforts for documentation and thus substantial involvement by participating GPs, we conducted a pilot study to see whether such a study design was feasible.

**Methods:**

We used a mixed methods design with two study arms in a sample of teaching GPs of the University Halle. The quantitative arm evaluates participating GPs and documentation of home visits. The qualitative arm focuses on reasons for non-participation for GPs who declined to take part in the pilot study.

**Results:**

Our study confirms previously observed reasons for non-response of GPs in the particular setting of home visits including lack of time and/or interest. In contrast to previous findings, monetary incentives were not crucial for GPs participation. Several factors influenced the documentation rate of home visits and resulted in a discrepancy between the numbers of home visits documented versus those actually conducted. The most frequently reported problem was related to obtaining patient consent, especially when patients were unable to provide informed consent due to cognitive deficits.

**Conclusions:**

The results of our feasibility study provide evidence for improvement of the study design and study instruments to effectively conduct a documentation-intensive study of GPs doing home visits. Improvement of instructions and questionnaire regarding time variables and assessment of the need for home visits will be carried out to increase the reliability of future data. One particularly important methodological issue yet to be resolved is how to increase the representativeness of home visit care by including the homebound patient population that is unable to provide informed consent.

## Background

Home visits are an integral part of general practice work in Germany. The current mean number of home visits by German general practitioners (GP) is approximately 25 per week [1]. There is evidence that average patient age and the frequency of medical home visits are positively associated [2]. Both national and international studies show that older age groups in particular (>75 years) utilize home visits more frequently [1,3,4]. This is important in the context of the growing proportion of the elderly in the German population [5] and the increasing prevalence of chronic conditions in older people [6]. This especially concerns chronic diseases related to longer life expectancy such as coronary heart disease, cancer, diabetes mellitus 2, and degenerative diseases [7]. In Germany, over two thirds (1.62 million) of patients in need of nursing care live at home. Of these homebound patients, over two thirds (1.07 million) are cared for exclusively by relatives and one third by relatives in conjunction with an home nursing care. [8]. This patient group, mainly characterized by multi-morbidity, comprises a high proportion of GPs’ home visits in Saxony [9].

According to the latest published data from Central Research Institute of Ambulatory Health Care in Germany (Zentralinstitut für kassenärztliche Versorgung), the number of home visits in Germany decreased approximately 40% between 1996 and 2003, although the percentage of visits to the older age groups has continuously increased [1]. In Saxony, the number of registered home visits by GPs has decreased over the last 5 years [10], a tendency that has been internationally observed for more than 20 years [1,11]. The decrease in routine (non-emergency) home visits has multiple causes such as better transport facilities for patients and improvements in communication techniques between homebound patients and doctors. As a consequence, the proportion of the urgent home visits by GPs has increased [1,12].

Although a growth in the elderly population results in a greater need for medical home care, the number of GPs in Germany is decreasing with fewer GPs available for home visits. Open questions regarding the organisation of primary care in ageing societies such as Germany thus remain: Do homebound patients receive sufficient medical care by GPs? Has the need for home visits in urban and/or rural regions changed? Concerning the expected reduction in the number of GPs, which components of home visits could be or are already delegated to health care assistants or practice nurses?

To answer these questions, we will be conducting a representative primary data collection concerning contents and organisation of GPs’ home visits over a period of 12 months in 2014/2015. We describe the aims of this future study as well as the current methodological feasibility study, which is the focus of this paper.

Organisation of home visits differs among GPs depending on organization of individual practices; additionally, the work week, accounting quarter and seasonal effects must also be considered. The data collection methods for most of the German and international studies regarding GPs’ home visits are based on secondary analysis of billing data coded by ICD-10 [1-4,11,13,14]. Because of limitations of ICD-10-coding in primary care, the description of work contents using the International Classification of Primary Care (ICPC) is more reliable [15-18]. Furthermore, billing data of home visits for the Association of Statutory Health Insurance (Kassenärztliche Vereinigung) do not include sufficient information about morbidity, diagnostics and therapy. They only include information about classification (urgent vs. routine) and time of the home visit (during or outside consultation hours). Thus, it is essential to gain primary data about home visits for describing content and organization of GPs’ home visits.

Prior to the planned main study we conducted a feasibility study to assess the viability of the study design. With regard to the response rate, the study explored willingness of GPs to take part in this more extensive documentation study compared to a one-time survey. Most of the published methodological studies reflect reluctance of GPs to comply only with postal or telephone surveys [19-26]. There are a few studies focused on recruitment of GPs in more extensive studies, such as randomised controlled studies [27-29] or other clinical intervention trials [30,31] or observational studies requiring greater workload by participating GPs [32]. The most frequently reported barriers by GPs were lack of time or insufficient personal resources for participation. Because our planned future study will require considerable efforts for documentation and thus substantial involvement by participating GPs, we carried out this pilot study to answer following group of questions:

A) Is it feasible to recruit GPs to participate in a more documentation intensive study? Is there a selection bias in terms of differences between the participating and non-participating GPs?

B) What was the likelihood of receiving completely filled out surveys without documentation errors (for example concerning date and duration of the home visit, travelling time). And lastly, what was the GPs’ practical experience with filling out study instruments and feasibility of doing this in the context of a home visit?

## Methods

Our feasibility study was coordinated at the Department of General Practice of the Technical University of Dresden and took place in 2012. Ethical consent was confirmed by the Ethical Commission of the Technical University of Dresden (EK 291082011). The study was conducted in cooperation with the Department of General Practice of the Martin-Luther-University of Halle/Wittenberg. To test the study design and data collection instruments, we used a sample outside of Saxony in order not to bias the Saxonian GPs scheduled to participate in the main study.

**Figure 1:**
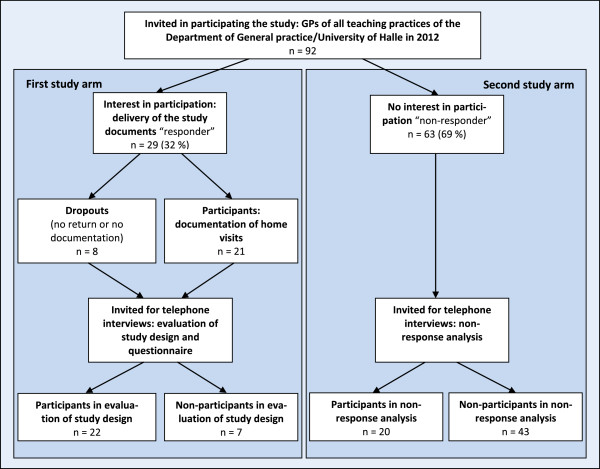
Flow chart of the feasibility study.

Based on the different objectives of the study, we used a mixed methods approach. This led to a design including two study arms (Figure 1). The first quantitative study arm was focused on participating GPs and documentation of home visits. The second qualitative study arm was focused on non-participants and reasons for non-participation. We invited all currently active teaching practices affiliated with the Department of General Practice of the University of Halle/Wittenberg to participate in our study. The postal invitation included cover letter, study information and response letter.

### First study arm: participating GPs

#### Documentation of home visits

Study materials were delivered personally by means of a facilitator visit to GPs who expressed their interest in participating. Study materials included instruction, semi-structured documentation sheets, patient information, content sheets, and a one-side questionnaire about demographic characteristics of GPs and practices as well as a form for expense allowance.

The study period lasted 3 months, starting in May 2012. Each GP was invited to document all home visits conducted in the period of one week. All patients who signed the consent sheet were included. According to additional agreement with the Ethical Commission of the Technical University of Dresden, patients unable to provide informed consent due to cognitive deficits were included if the care giver signed the consent sheet *and* the reason for cognitive disability was documented by the GP.

Depending on availability of GPs, the allocation of study week was done in consultation between GP and a member of the research staff of the cooperation partner aiming to have in minimum one study practice during every week of the study period. The GPs received a financial incentive of 3 Euro per documented home visit. The study documents (completed questionnaires and forms for expense allowance) were sent back in a prepaid envelope.

Study questions included concerning date and duration of the home visit, travelling time, sex, age and known chronic diagnosis of the patient, housing situation, kind of home visit, reasons and results for encounter/home visit, diagnostic and therapeutic decisions as well as assessment of the need for a home visit.

The questionnaires were checked for plausibility using SPSS 19.0. Reasons for and results of each encounter were coded manually into a mixed codification based on ICD-10 and ICPC-2 by two coders independent of each other. Discrepancies between the codes were documented and a third person decided after consulting each coder. Collection and analysis of the patients’ data were done anonymously. Statistical analysis focused on feasibility of study materials, such as analysis of missing data or tendencies in patterns of answers. Although there is no consensus at which cut off the missing data will bias statistical analysis [33], we decided based on our limiting sample size to analyse the pattern of incompleteness for variables in case of an amount of 10% missing data [34]. Additionally, items concerning contents of home visits were analysed to determine the appropriateness of the questionnaire for documenting home visits.

#### Interviews for evaluating study design and questionnaire

For assessing study procedures and questionnaires a short (5 to 10 minutes) semi-structured telephone interview was conducted shortly after (first contact up to maximum 14 days) the week of investigation to minimize the recall bias. The interviews were based on a guide using techniques of cognitive pretesting [35]. To enable interviewees to develop extempore narration, such as descriptive and argumentative communication schemes, a topic guide with open questions was used. The main focus was on experiences of participating in the study and comprehension and appropriateness of the questions. Therefore methods of comprehension probing were utilised. Each interview was recorded by telephone recording set, provided the GP agreed with recording. After transcription, a qualitative content analysis of the answers was conducted. This analysis (according to qualitative content analysis by Mayring) aimed to classify the content of the interviews to indicate pertinent categories such as comprehension of questions, biases or problems in documenting home visits [36]. Data were pseudonymously recorded and analysed.

### Second study arm: non-participating GPs

To reasons for non-response, a short (5 to 10 minutes) semi-structured guideline-based telephone interview was conducted. GPs who did not participate in the study were asked to participate in the non-responder analysis exploring barriers to participate. Interviews were recorded and analysed in the same manner as the study design interviews, with pseudonymous recordation and analysis of data. If GPs opted not to participate in the telephone interview, their reasons for unwillingness were asked and documented.

To compare socio-demographic data and organisational features of the different subgroups (participating documenting GPs, non-participating GPs, and GPs who dropped-out (Table 1)), we used exact tests (chi^2^ test, Fisher test, median test) due to the small sample sizes of the subgroups.

**Table 1:** Description of participating and non-participating GPs

**Parameters**	**Statistics**	**Participating GPs**		**Non-participating GPs**		**Total (n = 92)**	**Tests on differences**
		**GPs documenting home visits (n = 21)**	**drop outs (n = 8)**	**GPs interviewed for non-response analysis (n = 20)**	**GPs with no response (n = 43)**		
Female^$^	N	14	4	18	32	67	exact chi^2^ test: p ≥ 0.05
Urban region (>200000 inhabitants)^$^	N	12	4	13	32	61	exact chi^2^ test: p ≥ 0.05
Single practice^$^	N	18	8	14	35	77	exact chi^2^ test: p ≥ 0.05
Age^$$^	Median	53	46^#^	54	--	--	exact median test: p ≥ 0.05
Delegation of home visits^$$^	N	13	--	16	--	--	Fisher’s exact test: p ≥ 0.05
Estimated number of home visits per week^$$^	M ± SD	20 ± 11	12 ± 3^#^	17 ± 16	--	--	two sample t-test: p ≥ 0.05
Estimated percentual number of patients ≥ 65 years^$$^	M ± SD	56% ± 17%	--	45% ± 19%	--	--	two sample t-test: p ≥ 0.05
Estimated number of nursing home residents^$$^	Median	33	--	24	--	--	exact median test: p ≥ 0.05

^#^Based on telephone interviews with 3 non-responders.

^$^Administrative data from Department of General Practice/University Halle.

^$$^Data from questionnaire (documenting GPs) or interviews (non-participating GPs and drop outs).

– no data collected.

## Results and discussion

### Recruitment and participation rates

After postal invitation, 29 of 92 GPs (32%) confirmed their interest in participation per fax. Twenty-one of 92 GPs (23%) documented their home visits during the allocated week and sent the completed survey instruments back. One participating GP interrupted the documentation after 2 home visits. The response rate of 23% is in line with response or participation rates (ranged between 19 and 36%) of other studies that recruited German teaching general practices [31,37,38].

Four GPs did not start the documentation because of personal reasons (2) and/or disagreement with obtaining patients’ consent (3). Two GPs indicated that they documented home visits and assessed study design and instruments in the telephone interview, but failed to send back documentation materials. Two GPs were could not be reached for follow up for unknown reasons. There were a total of eight dropouts with regards to documentation. Documenting GPs and dropouts were asked to assess study design and instruments. 22 telephone interviews were conducted (19 documenting GPs, 3 dropouts) as outlined in the first study arm of Figure 1. The response rate of responders was 76%. This higher response rate is almost the same as those of follow-up surveys [27,39].

All non-participants were contacted by phone for invitation for telephone interviews to analyse reasons for non-response. 20 of the 63 non-responders (30%) participated (see Figure 1, second study arm). The response rate was in line with another German study assessing reasons for non-response or non-participation in a follow-up [40].

### Differences between participating GPs and non-participating GPs

Comparing the descriptive data of the participating documenting (Table 1, column 3) and non-participating GPs (Table 1, column 5/6) there were no significant and visible differences regarding type of practice and estimated number of home visits. Median age was similar in the groups of documenting and interviewed non-participating GPs. Some differences were nevertheless visible but not significant. Based on the small sample sizes only strong effects could be measured. In comparison to non-participating GPs, documenting GPs tended to care for an older patient population (≥65 years) and less for nursing home residents. Documenting GPs delegated their home visits to other medical professionals less frequently than interviewed non-participating GPs. There were on average ten percent less GPs from urban regions in the subgroup of documenting GPs compared to non-participating GPs as well as to the total sample. This was also observed in a former study, where significantly more GPs from rural regions in Saxony participated [9]. This association is not consistent with international data that showed no impact of urban milieu and response rates among physicians of 17 different specialty areas [41]. However, differences in organisation of medical care based on urban rural differences in the context of differing health care systems limit this international comparison.

Drop outs were characterized by younger age, more single practices and smaller number of home visits as well as they were more often from rural regions than the other subgroups. But based on the small dropout sample size of three, the analysis is quite limited.

### Reasons for participation in the study

Most frequently reported reasons for participation by the GPs were: interest in the topic of the study (5), to support research in primary care (4), to influence health policy in a way that will improve their working conditions (4) and to support family medicine in general (5).

Majority of the GPs (13 of 21) stated that the incentive of 3 Euro we gave in the study was not a motivation to participate and would have equally been likely to participate without monetary incentive. Regarding the question whether the incentive could motivate GPs that are indecisive to participate, several GPs assessed a monetary incentive of 3 Euro per documentation sheet as ridiculous and not attractive for GPs. One GP assessed it as a small incentive, but argued that GPs who do not want to support a study would also not do it for a greater incentive. Five GPs stated that the interest in the study/topic is crucial and not the financial incentive. This is not in accordance with most of the studies analysing factors that influence response rates of physicians. Independent on study designs, the majority of these studies showed an association between even modest monetary incentives (between 1 and 5 US Dollars) and higher response rates when compared with no incentive [19,25,26]. One possible explanation is that teaching practices (our study sample) are more motivated to take part in research studies than physicians that are not connected to the academic medicine.

In summary, reasons to participate beyond work-related and personal factors include personal or professional interest in a topic and the perceived relevance in research in primary care [19,27,40,42].

### Reasons for non-participation in the study

The primary reasons given for non-participation were lack of time or the heavy burden by documentation (15 von 21).These are well documented main reasons for GPs non-responding in one-time surveys as well as in longer lasting clinical trials [19,22,29-32,38,40].

Three GPs stated that the study design did not fit with their home visit schedules. One of them conducted planned home visits every six weeks. The two other GPs assessed that they did not have enough home visits for the study. Two GPs who were willing to participate forgot to confirm their interest because they misplaced the request in a pile of paperwork. These reported reasons show potential for improving the response rate by presenting more detailed study information for example while a personal delivery of the study instruments in the practice [27]. Delivering an information sheet to the practice staff, including workflow instructions combined with a checklist, could support the implementation of the study while decreasing drop-out rate [43,44].

Reported reasons for physician drop-out in our study were 1) personal reasons such as illness and 2) unwillingness to obtain patients consent. GPs argued that it could disrupt the doctor-patient relationship and to explain the information of the content sheet and to get the consent (sign) was too complicated and took too much time. The recruitment problem based on obtaining patient consent is not new and inherent in clinical research [28,29].

### Feasibility of the study design

Two of 21 documenting GPs did not document at allocated week because they forgot to bring the study instrument or the consent sheet with them to the home visits. In these cases we arranged another documentation week with both GPs and they did the documentation at this later date.

The majority of participating GPs (15 of 21) confirmed in principle the feasibility of documentation of all home visits of a whole week. Some GPs stated that it was feasible and reasonable if they got a good preparation by their practice nurses. This confirms the non-negligible impact of medical staff on feasibility of research with GPs. Some of the GPs also pointed to the time-consuming process of documentation home visits over a whole week period. Most frequently reported reason was the effort to obtain the consent by the patients.

**Figure 2:**
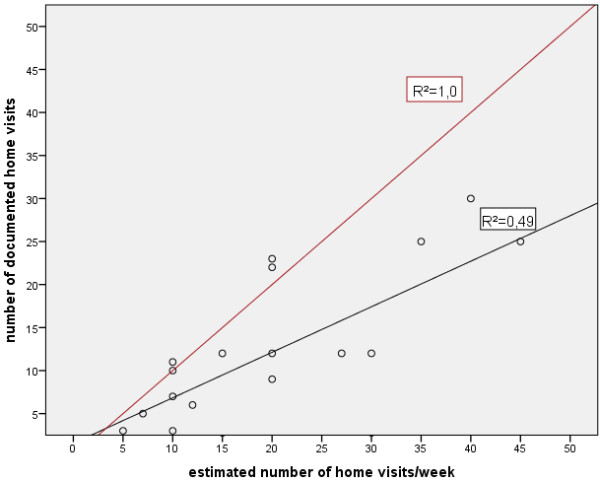
**Correlation between estimated number of home visits and documented number of home visits.** Detailed legend: (GP that interrupted documentation was excluded).

Comparing the estimated number of home visits (mean 20 ± 11) with the documented number of home visits (mean 12 ± 9), there seems to be a gap between estimation and documented reality. Majority of GPs documented significant less home visits than they conduct in average per week (see Figure 2, the points under the red line that mention the optimised scenario describing that number of documented correspondent to estimated number of home visits).

In the interviews, eleven GPs confirmed that they documented all home visits of a week whereas seven GPs pointed out problems in documenting all home visits of a week. Three GPs did only document home visits of four days because of a free day for the team in the allocated week. One GP forgot the documentation in cases of urgency home visits. Three GPs reported that they did not document all home visits because of the missing patients’ consents. In summary, the answers in the interviews validated and gave explanation to the observed under-representation of documented home visits in our study.

Documentation of home visits was primarily done by GPs conducting the home visits (n = 20). In one case the health care assistant did the documentation although the GP conducted the home visits. In another case health care assistant and GP shared the documentation depending on the person conducting the home visit.

**Figure 3:**
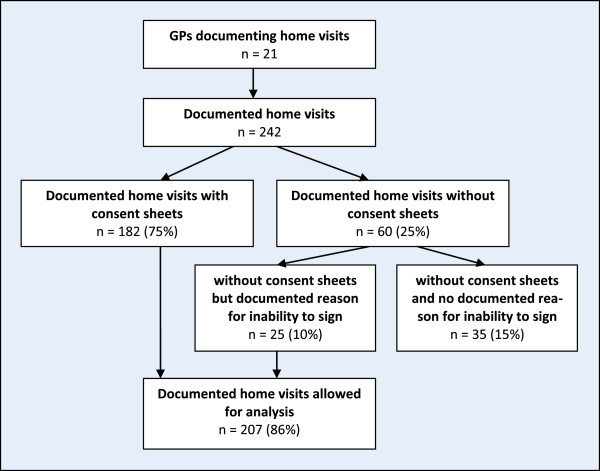
Documentation of home visits.

Problems in obtaining patients consent were predominant in home nurse residents where a visible number of patients are unable to provide informed consent due to cognitive deficits. Some GPs documented home visits of these patients without consent. Based on the explained problem with the consent, a GP constrained the feasibility of the study only on home visits that took place in private homes. Consequently, he did not document his home visits at home nurse residents. GPs 244 patients had a survey instrument completed during a home visit. 45 of these survey instruments were excluded because of missing consent form (see Figure 3). Besides these “visible” missing patients there were an unknown number of non-documented patients that were unable to provide informed consent due to cognitive deficits. These missing values are an important source of bias when describing characteristics of home visits.

### Assessment of study instruments

The one-side questionnaire about demographic characteristics of GPs and practices was send back by 20 of 21 GPs. An oral (at the end of the telephone interview) and a postal reminder (including the questionnaire) were mentioned to the one missing GP, but he did not send it back. One question except, all questions were fully answered and answers seems to be plausible. Two GPs gave no answers regarding the question of number of patients who live in nursing homes.

All GPs assessed the two-sided semi-structured documentation sheets as comprehensive and easy to handle. Only one GP expressed the wish concerning a shorter questionnaire. Another GP asked for more detailed information in the instructions regarding documenting travelling time in case of home visits in nurse home residents. Several GPs pointed to doubling of contents in the questions concerning results for encounter and decision on therapies. All of these arguments highlight the importance of using a short questionnaire. The impact of shorter questionnaires on response rates in primary care setting is well assessed [25,26,41,45]. But it is to consider, that a short questionnaire automatically includes a limitation of number of obtaining information.

Missing rate in answers was below 5 percent in the majority of the questions (concerning date and duration of the home visit, travelling time, sex and age of the patient, housing situation, kind of home visit, assessment of the need for a home visit). Somewhat higher missing rates were observed concerning care level (6%), assessment of social support (9%) and time of home visit (11%). Analysis of the pattern of incompleteness for time of home visits showed significant accumulation of missing data in two GPs (16 of 23 missing data). The remaining missing data were distributed over four GPs.

Analysing the GPs with accumulated missing data, there were no unusual characteristics compared to the other GPs. In the interviews only one of both GPs reported problems in filling the documentation sheets that concerned problems with remembering traveling time. But there was no explanation concerning problems with documenting the time of the home visit.

Several interviewed GPs also reported difficulties with recording and documenting time variables because of recall problems. In conclusion, instructions for GPs participation should point out more clearly how GPs should document these variables to avoid recall problems. GPs reported no problems in documenting patients’ morbidity, reasons and results for encounter as narrative answers.

**Figure 4:**
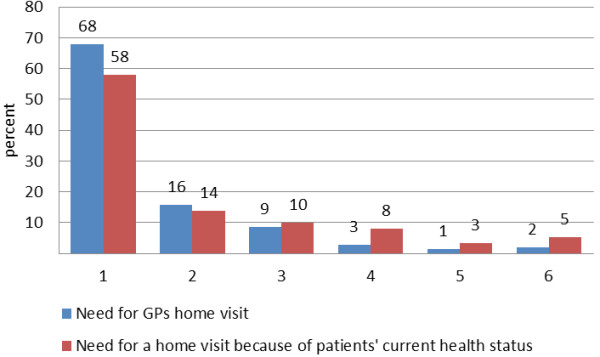
**Assessment of need for home visits.** Detailed legend: (1 = applies completely to 6 = does not apply).

The questions concerning assessment of the need for the documented home visit showed strong tendencies (floor effect described by a positively skewed distribution) in answers (see Figure 4). This allows several interpretations. 1) The data reflect from GPs point of view the need for home visits that seems to be very high. 2) Answers could be influenced by social desirability in this way that GPs who decided to conduct a home visit would not question their own behaviour. Asked for the general comprehensibility of the questions in the documentation sheets, one GP focused on these questions. This GP pointed out that his decision of conducting a home visit automatically includes the need for a home visit. Furthermore this GP explained that there are only two alternatives in need for home visits: *yes* or *no* and nothing in between.

Comparable, but inverse patterns of distribution were observed regarding the questions 1) whether the patient could also visit GPs’ practice and 2) whether a health care assistant or practice nurse could also conduct this home visit. In summary, the inversed patterns of response confirm the answers in the question of the need for GPs’ home visit.

The majority of the GPs did not miss basic topics regarding home visits. One GP who stopped her documentation after two home visits commented that the documentation sheet does not collect information on the narrative counselling of patients, which is the most important and time-consuming part of home visits. Additional recommendations included: focusing more on content and on counselling chronic and palliative patients, dividing acute and urgent home visits, and analysing contents of home visits after a hospital stay.

### Strengths and limitations of the study

To our knowledge, this is the first study focusing on the methodological feasibility of obtaining data on GPs’ home visits. From a methodological point of view, the pilot study sample was useful to assess whether the study instruments were feasible. Because the population of the main study will also include non-teaching GPs, the results regarding home visits might differ between this study pilot and our future planned study group. We found that the role of monetary incentives did not play a major role in participation. This may be because the monetary incentive was too low and thus not an actual factor in GPs decision to participate. Other factors involved the GPs decisions to participate are most likely a reflection of the intangible factors stemming from the longitudinal relationship of the GP teaching practice network affiliated to the University Halle.

## Conclusion

The results of our feasibility study provide evidence for improvement of both the study design and the study instruments in order to effectively conduct a documentation-intensive study of GPs doing home visits.

In terms of study design, difficulties with adherence to the pre-allocated documentation week underline the importance of keeping personal contact with and on-going reminders for participating GPs. Documenting all home visits over a week period was determined to be feasible by GPs in general. However, several factors influenced the documentation rate of home visits resulting in a discrepancy between the numbers of documented versus conducted visits. The most frequently reported problem was related to obtaining patient consent, especially when patients were unable to provide informed consent due to cognitive deficits. Additional ways of including this particular patient group is necessary to achieve adequate representativeness of homebound patient care.

In terms of study instruments, improvement of instructions and questionnaire regarding time variables and assessment of the need for home visit will be carried out to increase the reliability of future data.

Our study confirms previously observed reasons such as lack of time and/or interest for non-response of GPs in the particular setting of home visits [19,27,40,42]. In order to raise awareness in the topic and possible participation in our future main study, we will advertise our main study in local journals, relevant websites and at events for Saxonian GPs. In contrast to previous findings regarding modest incentives [19,25,26], monetary incentives did not a major role for participation in the context of the University Halle GP teaching practices we sampled.

## Abbreviations

GP: General practitioner; ICD-10: International Classification of Disease; ICPC-2: International Classification of Primary Care (Version 2).

## Competing interests

The authors declare that they have no competing interests. KV, AK, SB and AB are members of Saxon and German College of General Practitioners and Family Physicians (SGAM and DEGAM).

## Authors’ contributions

KV and AB developed the conception and design for the feasibility study. KV, ST and AB were involved in drafting the manuscript. AK, TF and SB revised the manuscript critically. All authors read and gave final approval of the final manuscript. Acquisition and analysis of the data was done by KV in cooperation with AK and TF.

## Pre-publication history

The pre-publication history for this paper can be accessed here:

http://www.biomedcentral.com/1471-2296/15/87/prepub
